# A generative model of the crab cardiac neuromuscular system with modulation

**DOI:** 10.1186/1471-2202-12-S1-P22

**Published:** 2011-07-18

**Authors:** Estee Stern, Keyla García-Crescioni, Mark W Miller, Charles S Peskin, Vladimir Brezina

**Affiliations:** 1 Department of Neuroscience, Mount Sinai School of Medicine, New York, NY, USA; 2 Institute of Neurobiology, University of Puerto Rico Medical Sciences Campus, San Juan, PR, USA; 3 Courant Institute of Mathematical Sciences and Center for Neural Science, New York University, New York, NY, USA

## 

The neurogenic crab heart is driven by the cardiac ganglion (CG), a central pattern generator that is embedded within the heart itself. The CG and the heart muscle form a complete closed-loop neuromuscular system. The bursting spike pattern generated by the CG drives the contractions of the muscle. These contractions, as well as previous spiking, then modify future spike generation. The system is extensively regulated by numerous neuromodulators. Here, we present a model of the complete system, with and without modulation.

We have modeled the system, at the level of spikes and contractions, as three processes: (1) the production of the contractions by the CG spikes, (2) the dependence of the CG spikes on the contractions, and (3) the dependence of the CG spikes on their own history. Using a system-identification method that we have developed [1], we first characterized each process separately from experimental data collected in the blue crab, *Callinectes sapidus*. Process (1) was characterized from spike-elicited contraction data in terms of three functions: *K*, the single-spike contraction kernel, *H*, a history kernel, and *F*, a static nonlinear function. These three functions successfully predict the contraction response to arbitrary spike trains. Using a modified version of the same method, we then characterized processes (2) and (3) together from data in which the CG spike pattern was recorded in response to stretches of the muscle. This gave three additional functions: *K’*, a kernel describing the effect of muscle length change on the generation of CG spikes, *H’*, a kernel describing the effect of a CG spike on the generation of future CG spikes, and *F’*, another static nonlinear function. Finally, we repeated all of these steps using data collected in the presence of modulators to find, in each case, six modified kernel functions.

**Figure 1:**
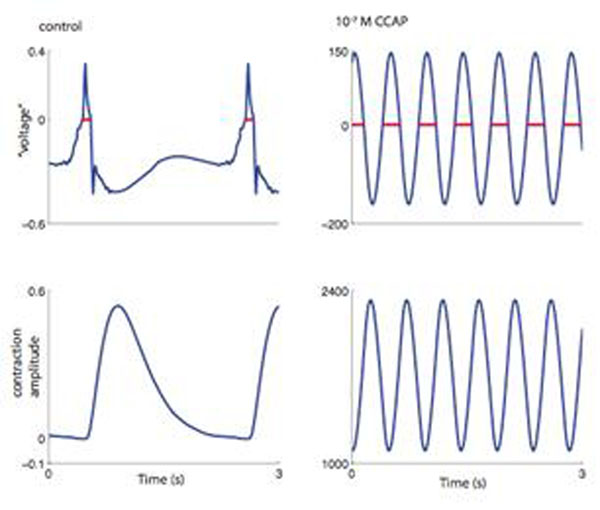
An example of the activity of the generative model. The left column shows the activity when the six functions used in the model were obtained from unmodulated data. The “voltage” is the sum of the operations of the functions *K’* and *H’* that can be roughly interpreted as reflecting the membrane voltage of the spiking neuron. The red dots indicate the spike times (grouped into bursts). The corresponding contraction amplitude is shown below. The right column shows the analogous results when the unmodulated functions *K*, *H*, and *F* were replaced with functions *K*, *H*, and *F* obtained from data with 10^-7^ M CCAP.

Combining the six unmodulated, or modulated, functions then gave a generative model of the complete cardiac system. This model exhibits a number of modes of spike generation, and resulting contraction, including multiple bursting modes, tonic firing, and no firing. It is likely that the modulators take the system from one mode to another. An example of a modeled shift induced by the addition of a modulator is shown in Fig. 1, where the addition of crustacean cardioactive peptide (CCAP) shortens the cycle period by ~75%. The model can be used to better understand the global actions of the modulators and how their multiple actions allow the cardiac system to respond to physiological demands in a robust, yet flexible manner.
